# Bioelectrical impedance vector analysis in obese and overweight children

**DOI:** 10.1371/journal.pone.0211148

**Published:** 2019-01-24

**Authors:** Beatriz de-Mateo-Silleras, Sandra de-la-Cruz-Marcos, Laura Alonso-Izquierdo, Mª Alicia Camina-Martín, José Manuel Marugán-de-Miguelsanz, Mª Paz Redondo-del-Río

**Affiliations:** 1 Department of Nutrition and Food Science, Faculty of Medicine, Valladolid University, Valladolid, Spain; 2 Department of Pediatrics, Hospital Clínico Universitario, University of Valladolid, Valladolid, Spain; Swinburne University of Technology, AUSTRALIA

## Abstract

**Introduction:**

BMI is the most commonly used indicator to evaluate overweight and obesity, but it cannot distinguish changes in body composition. Over recent years, it has been demonstrated that bioelectrical impedance analysis (BIA) is a more accurate method for analyzing body composition. Bioelectrical impedance vector analysis (BIVA) has revealed its effectiveness as an indicator of nutritional status and hydration.

**Objective:**

To assess the usefulness of bioimpedance analysis on the study of body composition in a group of children with overweight and obesity.

**Materials and methods:**

Cross-sectional observational study. The anthropometric parameters of 167 (79 were older than 12 years) overweight and obese children were recorded. Their body composition was analyzed using BIA and BIVA, and was classified based on different criteria. Concordance was analyzed (intraclass correlation coefficient, Bland-Altman analysis and weighted Kappa coefficient). The BIVA of the subgroups was compared using the Mahalanobis distance and Hotelling’s T^2^. Statistical significance was considered for p<0.05.

**Results:**

The BMI revealed that the majority of the assessed subjects were obese, although 12% had a normal percentage of fat mass (%FM). The classification by Z-BMI and Z-%FM significantly discriminate between subjects with different levels of adiposity. In children over the age of 12, the classification of fat mass index also discriminates significantly between obesity and non-obesity. As anticipated, in the tolerance ellipses, most of the individual vectors were situated in the left lower quadrant.

**Conclusions:**

BIVA reflects differences in the bioelectric patterns of children who are classified as being overweight or obese (BMI) and who have different levels of %FM and FMI. BIVA permits a fast and easy monitoring of the evolution of the nutritional state and changes associated with body composition, and it identifies those children whose body compartments may be precisely estimated using traditional BIA methods.

## Introduction

Changes in lifestyle and eating patterns in developed societies have led to an increase in the prevalence of chronic, non-communicable illnesses such as overweight and obesity. The prevalence of obesity in children between 5 and 19 years increased from less than 1% (5 million girls and 6 million boys) to almost 6% in girls (50 million) and about 8% in boys (74 million) between 1975 and 2016. In addition, 213 million children and adolescents were overweight in 2016 [1]. Childhood obesity is currently one of the main public health problems of almost every country in the world.

Given the extent of this problem, it seems evident that the best way to tackle it is through prevention, and thus it is necessary to have valid tools and criteria to allow for early diagnosis. The WHO has defined obesity as a condition in which an excessive fat accumulation impairs health and wellbeing [2]. Therefore, to detect overweight and obesity, weight alone is not a valid variable, and it is necessary to use appropriate indicators to measure fat mass (FM). In childhood, body mass index (BMI) has been traditionally used as an indirect measure of adiposity. However, there is no consensus on the establishment of the cut-off points to detect excess adiposity and to diagnose children as being overweight or obese [3]. Furthermore, it is common for different countries and even different regions to establish their own anthropometric reference standards. To facilitate the comparison between different studies and to attempt to determine the actual prevalence of the problem, international standards are available [4–7], but these references differ in their methodology, source and the age of data as well as in the classification criteria.

The best effective way to assess adiposity is to measure fat mass. Among other methods now available to estimate adiposity, there are techniques such as dual X-ray absorptiometry (DXA), nuclear magnetic resonance (NMR), computed tomography and ultrasounds [8]. In addition to the high cost of some of these methods, many of them cannot be used in childhood due to the characteristics of the technique, potential secondary effects, the need for collaboration of the subject and the lack of valid reference standards for children and adolescents.

In Pediatrics it has been recently used the four-compartment model to study body composition (BC), instead of the traditional two-compartmental method. This approximation, currently considered the reference model in studies of BC in children, combines independent measurements of bone mineralization, total body water, body density and weight [9]. This model requires the use of various techniques such as DXA, hydrostatic weighing or air displacement plethysmography, therefore it is mainly used in research. In clinical settings, it is more common to use methods that, although indirect, are simple, rapid, non-invasive, easy to carry out, cost-effective and free of risks for the patients. These methods include anthropometry and bioelectrical impedance analysis (BIA) [10].

The most commonly parameter used to evaluate overweight and obesity is the BMI, however, this indicator is not capable of determining whether changes in weight are due to FM, fat-free mass (FFM) or total body water content (TBW). Although the BMI has a high specificity to reflect adiposity [0.93 (confidence interval (CI): 0.88–0.96)], its sensitivity is not so high [0.73 (CI: 0.67–0.79)] [11], suggesting that almost a quarter of the children that are not classified as obese based on BMI may in fact have an excess of body fat. Therefore, the prevalence of obesity may be underestimated. FM also tends to be measured using skin fold measurements. However, this technique has many limitations [12] and it does not provide precise measurements of adiposity [13].

Up to date, studies have confirmed that bioimpedance analysis is more precise than anthropometry in determining BC [14]. This technique consists of introducing an alternating electrical current in the body and measuring the opposition (resistance) to the flow of current of the body. The most frequent type of analysis is the traditional BIA, which, like the anthropometry method, is based on a two-compartment model of BC. The traditional BIA requires the use of prediction equations to estimate TBW, FFM and FM. The precision of the estimate of FM depends largely on the suitability of the predictive model used [13]. The traditional interpretation of BIA has certain limitations in children, mainly resulting from the principles of the technique. It should be noted that in juvenile populations, the percentage of tissue hydration and the body proportions change with growth and development.

To solve these problems, the direct use of raw electric data obtained by the BIA analysis has been proposed, via the bioelectrical impedance vector analysis (BIVA) [15]. In this way, a vector of each subject is drawn (resistance/height-R/H- and reactance/height -Xc/H-) on a graph that represents the reference ellipses of the distribution of the normal values of R/H and Xc/H in a specific population (tolerance ellipses). This analysis provides a semi-quantitative assessment of cell mass and body water. Though it does not provide quantitative estimates of body water or cell mass, however allows the discrimination different liquid volumes (fluid overload and dehydration) and identifies variations in body cell mass. Various studies have demonstrated the efficiency of BIVA as an indicator of nutritional status and hydration in different populations during all phases of the life cycle, both in healthy and sick subjects [14,16–18]. Reference values are currently available for vectors of specific impedance, age and gender in healthy children [19,20]. In summary, BIVA may be a promising method to be used in the assessment of BC and its alterations in childhood.

The aim of this study was to assess the usefulness of bioimpedance analysis (BIA and BIVA) in the study of body composition in a group of children with overweight or obesity.

## Methods

A cross-sectional observational study was conducted, based on the review of clinical histories, including 167 participants suffering from overweight or obesity, aged between five and 18 years old. Participants were attended at the Pediatric Service, Hospital Clínico Universitario de Valladolid (Spain), between 2003 and 2015. The verbal/oral consent from the parents was obtained before the measurements. The study was approved by the corresponding Clinical Research Ethics Committee (CEIC), and all data were fully anonymized. Data were recorded from the anthropometric assessment and the analysis of body composition.

Anthropometric determinations (weight, height and abdominal circumference) were taken following the NHANES [21] and WHO [22] protocols. To do so, electronic scale and a stadiometer SECA (Hamburg, Germany) were used, as well as a flexible and non-extendible steel tape. All assessments were made by the same researcher. Body mass index was calculated; the Z-score of this indicator and its classification was established in accordance with the WHO criteria (overweight: Z-BMI≥1; obesity: Z-BMI≥2) [5]. The Z-score of the abdominal circumference (ACi) was calculated based on the reference values published by Arriba Muñoz et al. [23] for Spanish children and adolescents. Abdominal obesity was considered based on this Z-score, via a statistical criteria [low central fat mass (CFM) if Z-ACi≤ -2; normal CFM if Z-ACi > -2 < Z-ACi ≤ +1; high CFM if Z-ACi > +1< Z-ACi < +2; and obesity if Z-ACi ≥ +2).

A bioimpedance analysis (BIA) was conducted by the mono-frequency mode (at 50 kHz, 400 μARMS), using a tetrapolar electrode configuration, with an AKERN-Srl bioimpedance measuring device (Florence, Italy). The measurement method was standardized in accordance with the protocol used by Lukaski [24]. Based on the traditional BIA, body composition was estimated using the predictive model of Plachta-Danielzik et al. [25]. The standardized Z-score of %FM was calculated based on the reference data for the Caucasian population with ages ranging between four and 18 years old [25]. Subjects were classified according to the Z-%FM, considering both a statistical criteria (low FM if Z-%FM ≤ -2; normal FM if -2 < Z-%FM ≤ 1; high FM if 1< Z-%FM < 2; and obesity if Z-%FM ≥ 2), and also the International Obesity Task Force (IOTF) criteria [26]. For children over the age of 12, the body fat index (FMI) and free-fat mass index (FFMI) were calculated and they were ranked in accordance with the Bibiloni et al. criteria [27].

A semi-quantitative analysis was conducted for the body composition using the BIA vectorial modality (BIVA): the components of the impedance vector (R and Xc) were standardized by the height of the subjects (R/H (ohm/m) and Xc/H (ohm/m), respectively) and were represented on the R-Xc graph (abscissa axis: R/H; ordinate axis: Xc/H). Individual impedance vectors were compared with the distribution of vectors from the healthy reference population (tolerance ellipses at 50%, 75% and 95%) [19]. To compare the subgroups obtained from the nutritional classification, confidence ellipses were used, which were calculated with the mean impedance vectors for each group (software provided by A. Piccoli-BIVA Programs, release 2002).

The statistical analysis was carried out by the IBM SPSS Statistics 20.0 for Windows package. The parametric variables were described as mean (SD), and the variables that did not follow a normal distribution, as median (P25-P75). The normality of the variables was determined using the Kolmogorov-Smirnov or Shapiro-Wilk tests. Categorical variables were described as absolute and relative frequencies (n, %). The concordance between methods was assessed quantitatively using the intraclass correlation coefficient (ICC) and its 95% confidence interval at 95% (95% CI) and the Bland-Altman analysis. To study the concordance between different classifications, only children with excess fat mass have been considered. For so the Kappa index was determined and it was assessed according to the criteria of Landis and Koch [28]. To compare the BIVA between the subgroups established in accordance with the different nutritional status, the Mahalanobis distance (Md) was calculated and the Hotelling’s T^2^ statistic was used. Statistical significance was achieved with p<0.05.

## Results

**Fig 1:**
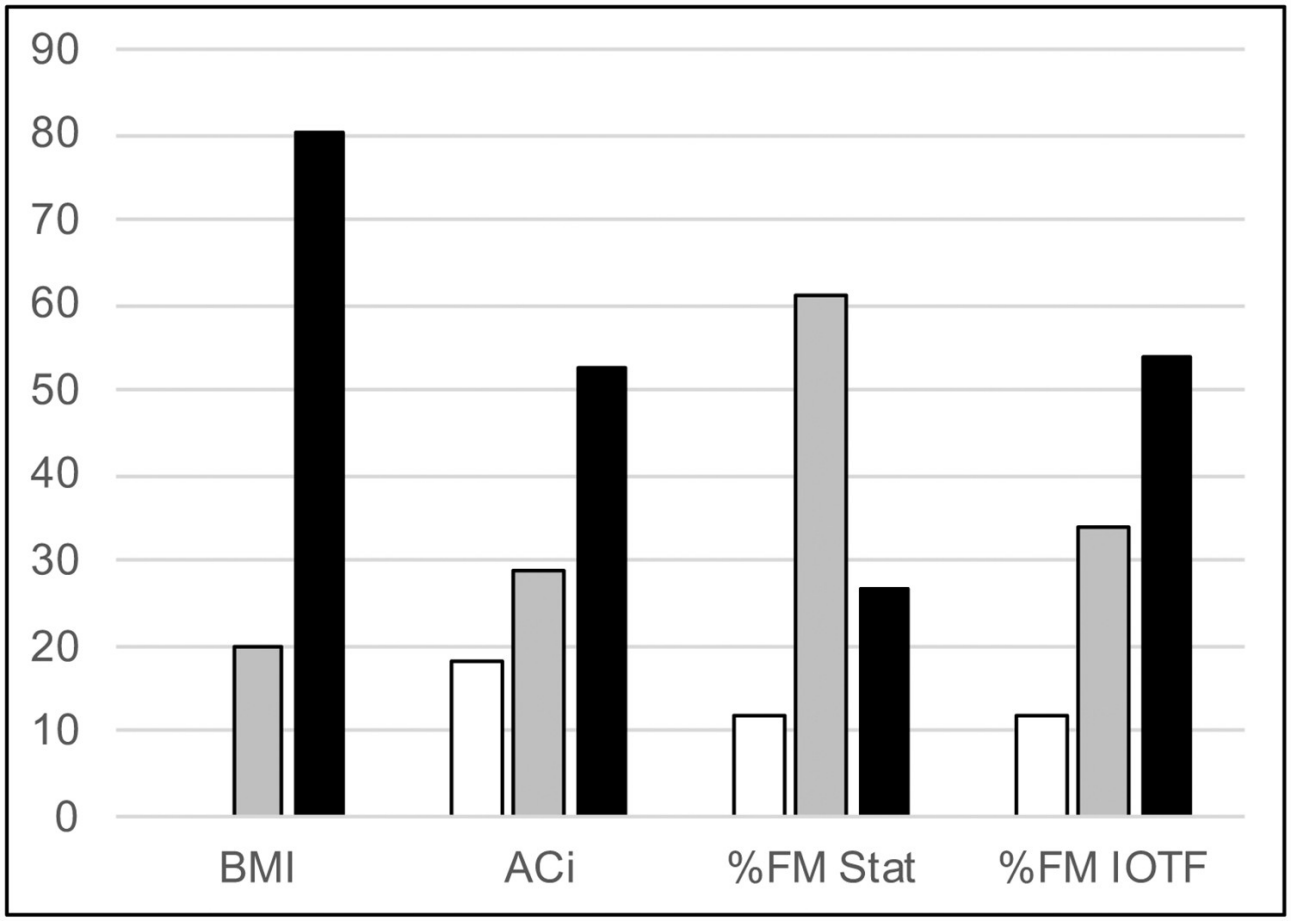
Nutritional classification of total sample based on the Z-BMI, the Z-abdominal circumference and the Z-% fat mass. The bars represent percentage of children. Black bars: obesity or abdominal obesity; grey bars: overweight, excess of central fat mass or overfat; white bars: normal central fat mass or normal fat. BMI: body mass index; ACi: abdominal circumference; FM: fat mass; Stat.: cut-offs according to the statistical criteria; IOTF: cut-offs according to the IOTF criteria.

The study includes a total of 167 subjects suffering from excess weight (69 boys (41.3%) and 98 girls (58.7%)), having a mean age of 11.4 (3.0) years. The Z-score of main anthropometric and body composition variables are shown in Table 1, and Fig 1 summarizes the classification of subjects based on these variables. The mean Z-BMI was 2.72 (0.86), indicating that most of the children were obese [134 obese (80.2%) vs. 33 overweight (19.8%)]. The mean Z-score of abdominal circumference was 2.65 (2.15); as a mean, the group of children suffered from abdominal obesity [abdominal obesity: 88 (52.6%); excess CFM: 48 (28.9%); normal CFM: 31 (18.4%)].

**Table 1:** Z-score of main anthropometric and body composition variables of total sample.

Variables	Z-score Mean (SD), N = 167	Classification and Z-score Mean (SD)					
BMI	2.72 (0.86)	**Overweight, N = 33 (19.8%)**			**Obesity, N = 134 (80.2%)**		
		1.67 (0.26)			2.98 (0.75)		
ACi	2.65 (2.15)	**Normal CFM, N = 31 (18.4%)**		**Excess CFM, N = 48 (28.9%)**		**Abdominal obesity, N = 88 (52.7%)**	
		0.49 (0.42)		1.41 (0.25)		4.01 (2.04)	
%FM	1.64 (0.73)	**Statistical criteria**			**IOTF criteria**		
		**Normal fat, N = 20 (12%)**	**Overfat, N = 102 (61.1%)**	**Obesity, N = 45 (26.9%)**	**Normal fat, N = 20 (12%)**	**Overfat, N = 57 (34.1%)**	**Obesity, N = 90 (53.9%)**
		0.23 (0.82)	1.58 (0.25)	2.40 (0.26)	0.23 (0.82)	1.39 (0.16)	2.10 (0.36)

BMI: body mass index; ACi: abdominal circumference; CFM: central fat mass; FM: fat mass; Statistical criteria: cut-offs according to the statistical criteria; IOTF criteria: cut-offs according to the IOTF criteria.

The mean Z-score for %FM of the total sample was 1.64 (0.73). The Z-%FM of the subjects, based on their classification by BMI, was 1.14 (0.60) in the subjects ranked as overweight, and 1.76 (0.71) in the obese subjects.

Considering Z-%FM classification with the statistic criteria, only 12% (N = 20) of children had a normal %FM, 61.1% (N = 102) were overweight, and 26.9% (N = 45) were obese (Table 1 and Fig 1). The IOTF criteria [26] ranked those same children as having normal %FM but not the remainding: overweight (34.1%, N = 57), and obesity (53.9%, N = 90) (Table 1 and Fig 1). That is, almost half of the children that were classified as being overweight based on statistical criteria are considered obese according to the IOTF criteria.

**Fig 2:**
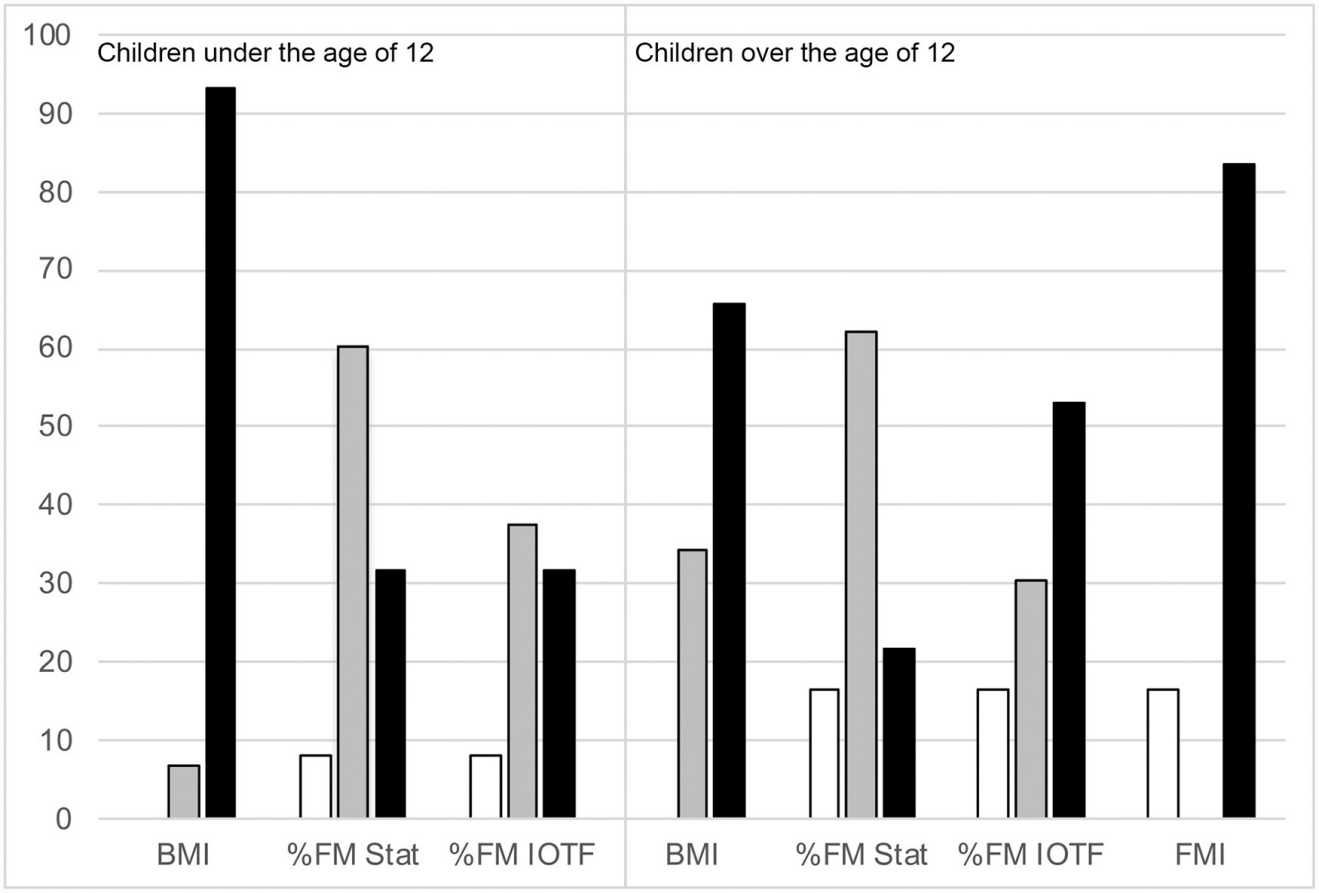
Nutritional classification based on the Z-BMI, the Z-% fat mass and the fat mass index in stratified sample (children under the age of 12 and children over the age of 12). The bars represent percentage of children. Black bars: obesity; grey bars: overweight or overfat; white bars: normal fat or non-obesity. BMI: body mass index; FM: fat mass; Stat.: cut-offs according to the statistical criteria; IOTF: cut-offs according to the IOTF criteria; FMI: fat mass index.

To estimate FMI and FFMI, the sample was stratified into two groups: children over the age of 12 [N = 79; 34 males (43%) and 45 females (57%)] and children under the age of 12 [N = 88; 35 males (39.8%) and 53 females (60.2%)]. FMI and FFMI were estimated in the first group, according to Bibiloni et al. criteria [27]. Table 2 shows the main anthropometric and body composition variables. Fig 2 reflects the nutritional classification based on the Z-BMI and Z-%FM in both groups, and on the FMI in children over the age of 12. Almost all children under the age of 12 were classified as being obese (N = 82; 93.2%) according to BMI, whereas in children older than 12 years, 27 patients (34.2%) were classified as being overweight, and 52 (65.8%) these were considered obese. The analysis of body composition in these groups was similar to that obtained from the total sample: the statistical criteria classifies more children as overfat, while the IOTF criteria considers a greater number of obese. According to the classification of FMI, 66 (83.5%) of these children were obese; therefore is, the FMI classifies all children as having an excess of FM as obese.

**Table 2:** Z-score of main anthropometric and body composition variables of stratified sample (children under the age of 12 and children over the age of 12).

Variables	Mean (SD)	
	Under 12 y. (N = 88)	Over 12 y. (N = 79)
Z-BMI	3.07 (0.86)	2.34 (0.70)*
Z-ACi	1.42 (1.12)	0.69 (0.70)*
Z-%FM	1.71 (0.78)	1.55 (0.66)^

*p<0.001 and

^p = 0.126 between groups.

BMI: body mass index; ACi: abdominal circumference; FM: fat mass.

**Fig 3:**
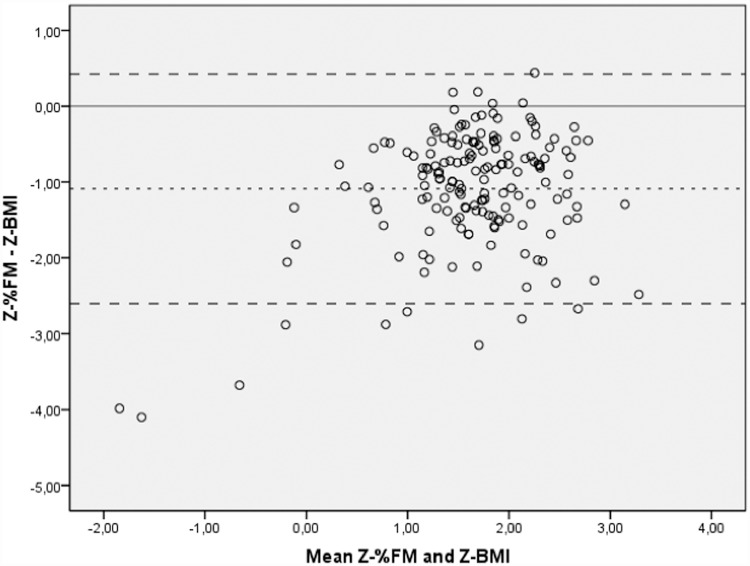
Bland-Altman analysis between the Z-BMI scores and the Z-%FM scores. The difference between the Z-scores from BMI and those of the Z-%FM was -1.088 (95% CI: -2.602 to 0.426), and there was only 1 case (0.6%) above the upper threshold the agreement interval, and 9 (5.3%) below the lower threshold.

A concordance analysis was conducted between the Z-BMI scores and the Z-%FM scores and a poor agreement was obtained [ICC of 0.276 (95% CI: -0.094 to 0.570) (p>0.05)], with greater disagreement for the lower mean values (Fig 3). The concordance between the nutritional classification based on both indicators (Z-BMI and Z-%FM, classified by statistical and IOTF criteria) is shown in Table 3. Using the statistical criteria, a 44.2% agreement was achieved; the Kappa index was 0.122 (95% CI: 0.055 to 0.191) (p = 0.004). Using the IOTF criteria the concordance was somewhat higher: Kappa = 0.241 (95% CI: 0.100 to 0.382) (p<0.001); agreement level: 68.0%) (Table 3).

**Table 3:** Contingency table of the nutritional classification based on the Z-BMI and the Z-% fat mass.

Z-%FM/ /Z-BMI	Statistical criteria		IOTF criteria	
	Overfat	Obesity	Overfat	Obesity
**Overweight**	21	1	16	6
**Obesity**	81	44	41	84
**TOTAL**	102	45	57	90

FM: fat mass; BMI: body mass index; Stat.: cut-offs according to the statistical criteria; IOTF: cut-offs according to the IOTF criteria. Results of the concordance analysis: (1) statistical criteria: agreement: 44.2%; Kappa index = 0.122 (95% CI: 0.055 to 0.191) (p = 0.004); (2) IOTF criteria: agreement: 68.8%; Kappa index = 0.241 (95% CI: 0.100 to 0.382) (p<0.001).

Table 4 shows that the concordance indexes between the Z-BMI scores and the Z-% FM scores, and between the classification based on Z-BMI and Z-%FM in both children under the age of 12 and children older than 12 years were also poor.

**Table 4:** Results of the quantitative and qualitative concordance analysis of stratified sample (children under the age of 12 and children over the age of 12).

Concordance indexes		Children under the age of 12	Children over the age of 12
**Quantitative: Z-BMI and Z-%FM**	**CCI**	0.246	0.292
	**(95% CI)**	(-0.090 to 0.568)	(-0.076 to 0.572)
	**p**	>0.05	>0.05
**Qualitative: classification Z-BMI and Z-%FM (Stat. criteria)**	**Kappa**	0.067	0.166
	**(95% CI)**	(0.006 to 0.128)	(0.039 to 0.293)
	**p**	0.093	0.03
	**Agreement**	40.7%	48.5%
**Qualitative: classification Z-BMI and Z-%FM (IOTF criteria)**	**Kappa**	0.175	0.337
	**(95% CI)**	(0.035 to 0.314)	(0.102 to 0.571)
	**p**	0.005	0.005
	**Agreement**	65.4%	71.2%

CCI: intraclass correlation coefficient; 95% CI: confidence interval at 95%; BMI: body mass index; FM: fat mass; Stat: statistical.

**Fig 4:**
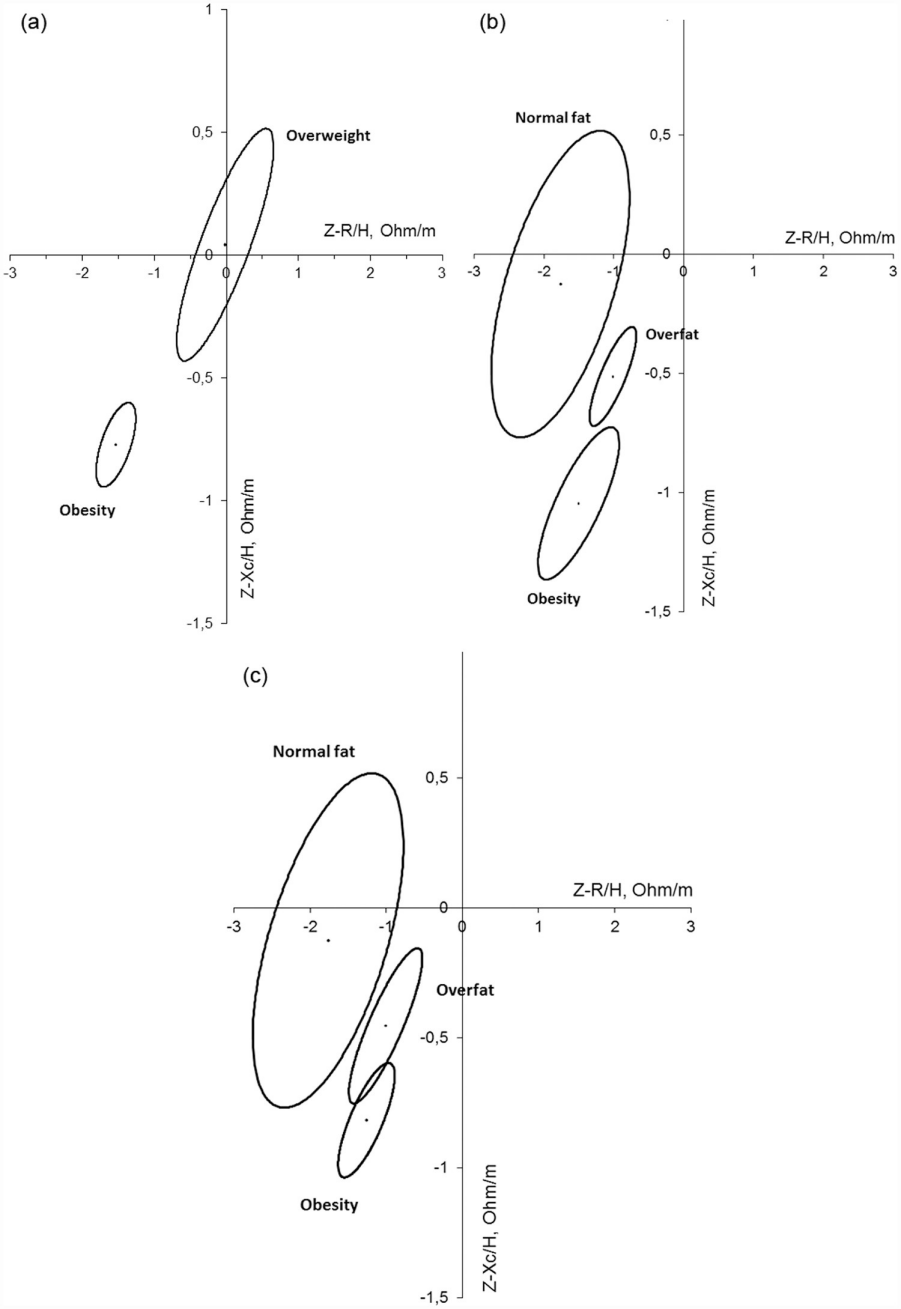
Confidence ellipses of the total sample based on the nutritional classification established form the Z-scores (a) of BMI, (b) of Z-%FM (ranked with the statistic criteria), and (c) of the Z-%FM (ranked with the IOTF criteria). (a) p<0.001 obesity vs. overweigth. (b) p<0.001 between groups. (c) p<0.001 nornal fat vs. the rest; p = 0.036 overfat vs. obesity.

**Fig 5:**
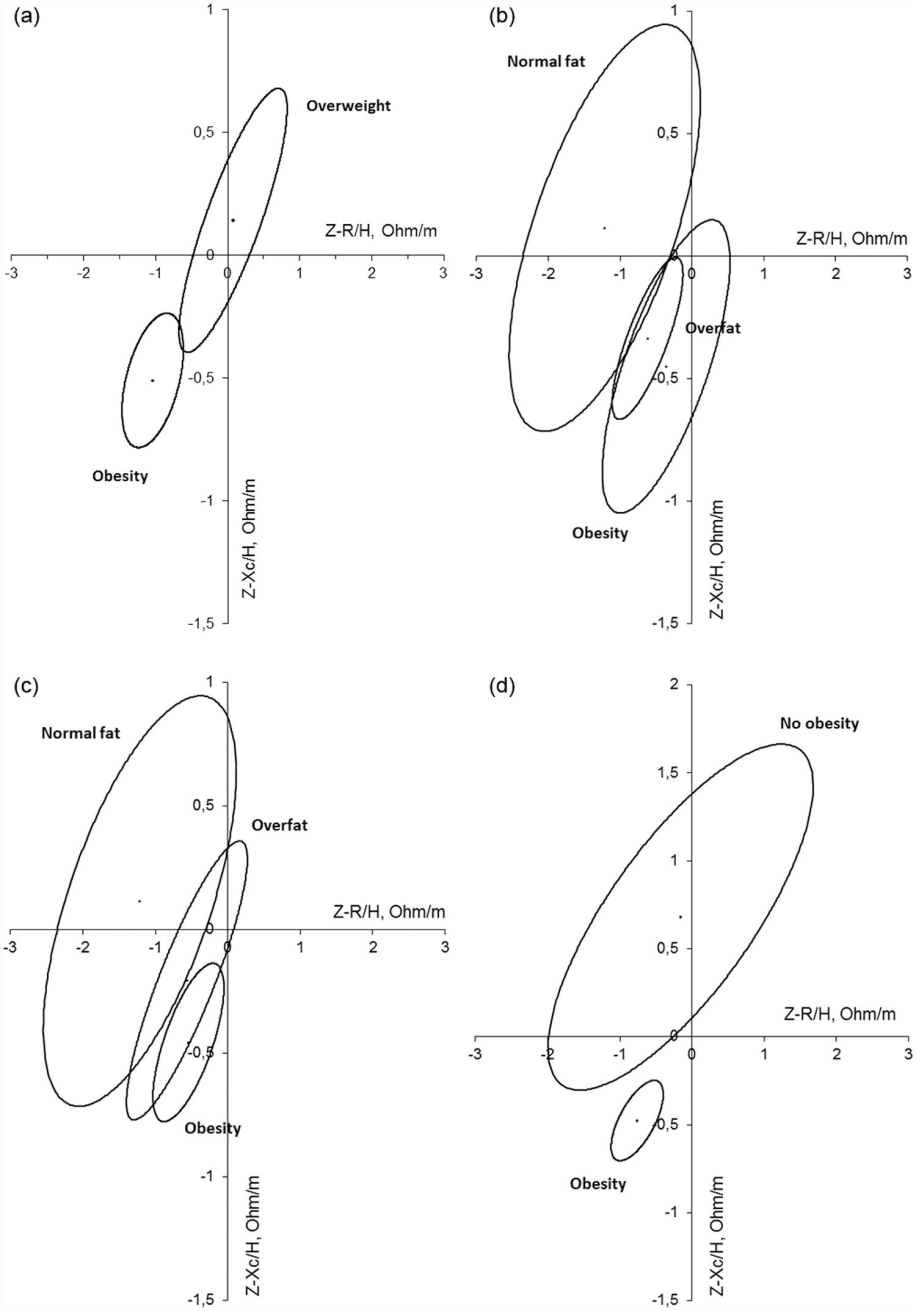
Confidence ellipses of individuals above the age of 12 based on the nutritional classification established form the Z-scores (a) of BMI, (b) of Z-%FM (ranked with the statistic criteria), (c) of Z-%FM (ranked with the IOTF criteria), and (d) from the nutritional classification based on the fat mass index. (a) p<0.001 obesity vs. overweight. (b) p<0.05 normal fat vs. the rest; p = 0.25 obesity vs. overfat. (c) p>0.05 between groups. (d) p<0.001 obesity vs. no obesity.

**Fig 6:**
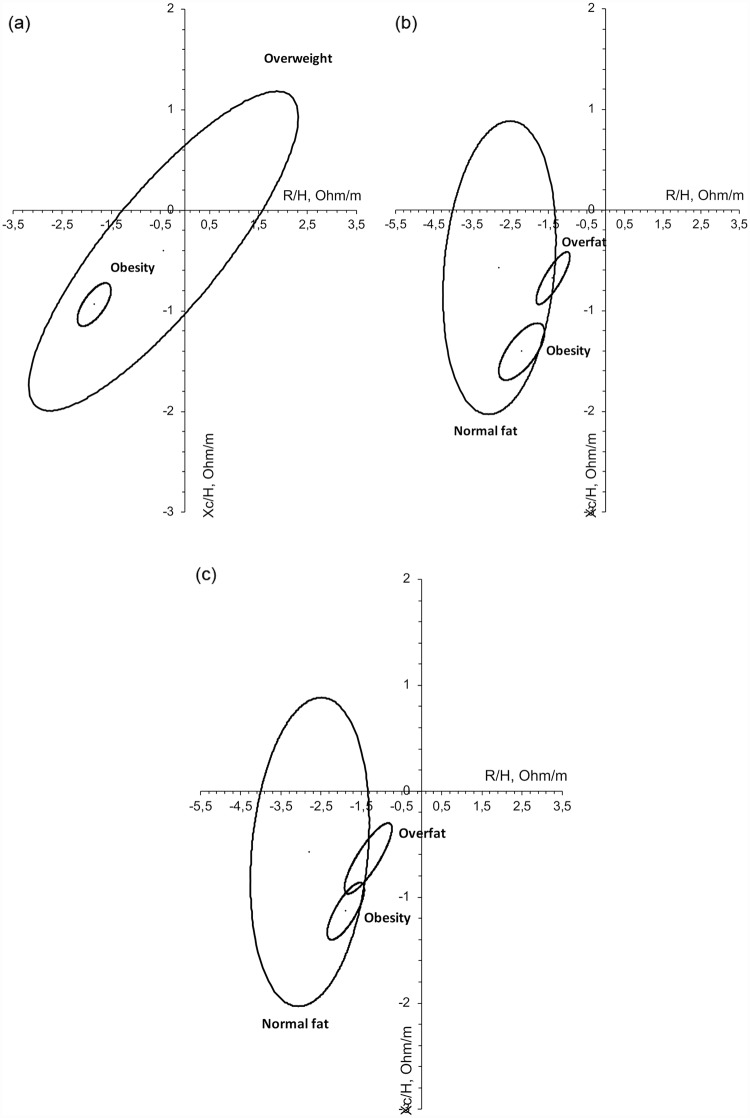
Confidence ellipses of individuals under the age of 12 based on the nutritional classification established form the Z-scores (a) of BMI, (b) of Z-%FM (ranked with the statistic criteria), and (c) of Z-%FM (ranked with the IOTF criteria). (b) p<0.001 overfat vs. obesity; (c) p = 0.001 overfat vs. obesity.

Finally, a vectorial analysis of bioimpedance was carried out. Confidence ellipses were created from the subjects based on the different nutritional status used in this study for the total sample (Fig 4): (a) Z-BMI, (b) Z-%FM–statistical classification criteria -, and (c) Z-%FM–IOTF ranking criteria-. The BMI and the classification of the Z-%FM with the statistical criteria discriminate in a statistically significant manner between subjects who are obese, overweight (a) and those with excess of fat mass and normal fat mass (b). With the classification of the Z-score based on the %FM with the IOTF criteria (c), the categories of obesity and overweight overlap slightly. In subjects over the age of 12 (Fig 5), the two classifications of the Z-%FM do not discriminate between different categories (b and c). However, the ranking of the fat mass index does discriminate between the categories of obesity and non-obesity (d). In children under the age of 12 (Fig 6) the confidence ellipses of the BMI (a) cannot be interpreted, since the overweight category includes few children (N = 6); and the same occurs with the ellipses of two classifications based on the Z-% FM. However, relating to overfat and obesity categories, the statistical criteria discriminates better both groups than the IOTF criteria. The same results are obtained with the total sample.

**Fig 7:**
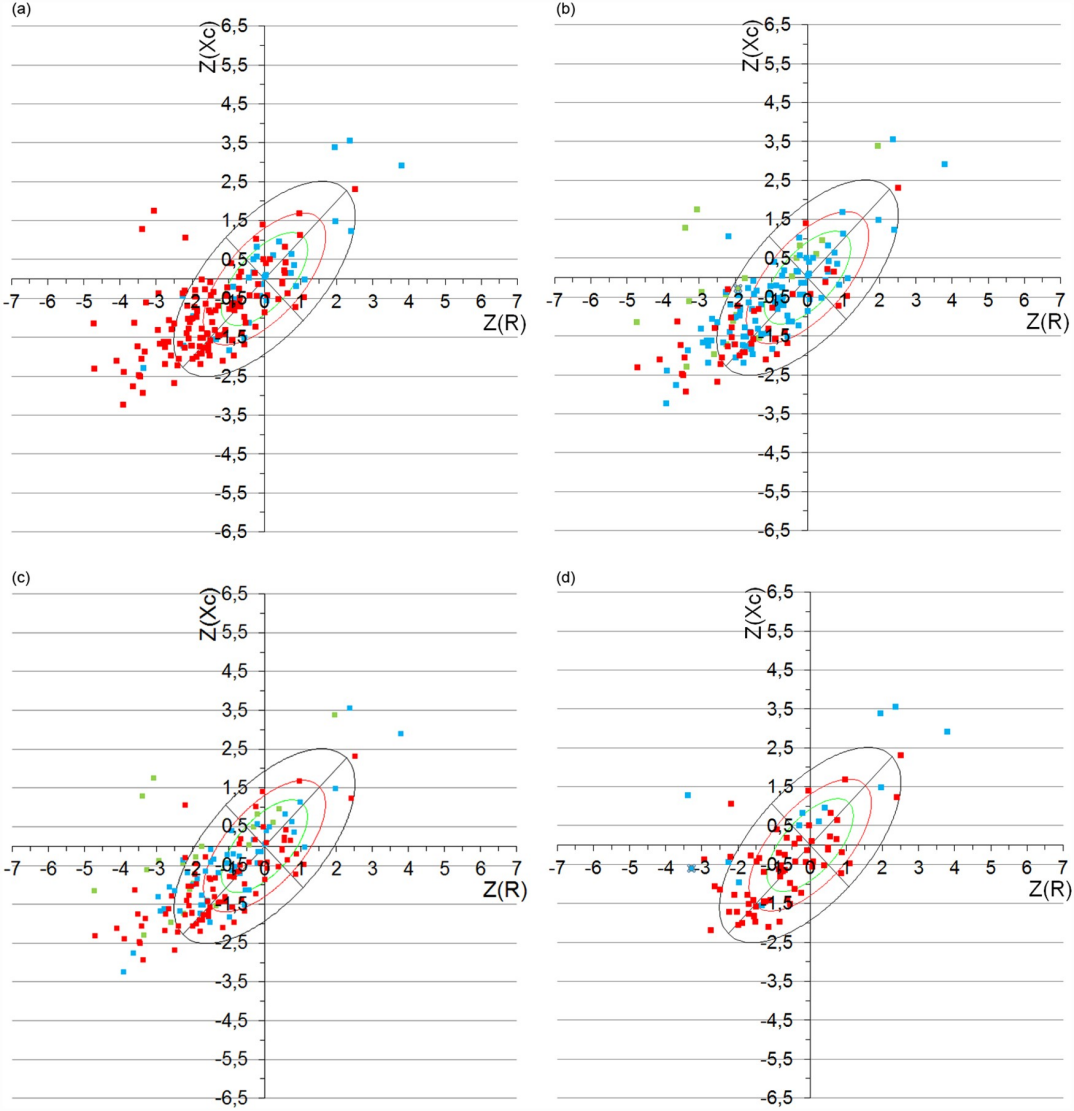
Tolerance ellipses based on the nutritional classification based on the Z-score of the (a) BMI, (b) Z-%FM ranked with the statistic criteria, (c) Z-%FM ranked with the IOTF criteria, in the total sample; and (d) fat mass index in children over the age of 12. Yellow dots: severe obesity; red dots: obesity; blue dots: overweight (body mass index), overfat (% fat mass) or no obesity (fat mass index); green dots: normal fat.

Tolerance ellipses were also constructed based on the normal classification from the BMI (a) and the Z-%FM with the two criteria used in the overall sample (b and c) (Fig 7). As expected, most of the individual vectors were situated in the right lower quadrant, suggesting an excess of adiposity. 19.8% of the subjects were outside of the tolerance ellipse of 95%, indicating abnormal impedance vectors. In children over the age of 12, 15.2% of them were situated outside of the tolerance ellipse of 95% (Fig 7(d)). 23.9% of the individual vectors of children under the age of 12 were situated outside of the tolerance ellipse of 95%.

## Discussion

This study assessed the nutritional status of a group of children and adolescents with overweight and obesity according to different methods such as the BMI or the waist circumference. Their body composition was also examined using bioimpedance (conventional and vectorial modalities). This work was proposed given the difficulty in making a correct nutritional assessment for this age group, since there is no international agreement as to the classification of obesity. Furthermore, for each method, it is difficult to find consensual criteria for classification. In addition to these limitations, differences also exist between the reference populations used in each country, or those derived from ethnicity.

BMI is a useful anthropometric indicator in epidemiology, given that it assesses the risk of being overweight or obese, but at an individual level, it has some major limitations. A high BMI reflects an excess of weight, which may be due to an increase in FM or FFM. Various studies [29,30] have observed that in many cases adiposity levels are quite distinct from expected, in accordance with the classification used based on BMI. This indicator has a high specificity, but lower sensitivity, therefore it is not useful in many subjects for the detection of an excess of adiposity [11,31,32]. In fact, many authors have questioned the utility of BMI for the diagnosis of obesity [33]. And, in childhood, BMI is unable to reflect upon the variations in body composition which may depend on the specific phase of weight-height development [34].

On the other hand, it is currently known that obesity is related to a low level of inflammatory state that favors the development of cardio-metabolic complications [35]. Recent studies have observed the importance of central adiposity, even from childhood, with regards to cardio-metabolic risk [36]. Thus, the waist circumference is a better indicator of the distribution of fat mass than BMI, since it defines the existence of accumulations of abdominal fat, which is associated with cardio-metabolic risk [37], and its value presents a good correlation with the FM estimated by DXA [38]. However, the use of the waist perimeter also has its limitations, since there is no consensus regarding the point where the circumference should be measured and given that there are no cut-off points indicating the excess of body fat in children and adolescents [39].

The existing limitations with regards to the anthropometric methods, especially in this age group, justify the need to carry out a body composition analysis. To evaluate adiposity, two indicators may be used: body fat percentage or, more recently, fat mass index (FMI). The main problem is the lack of agreement regarding the cut-off point of the %FM for the diagnosis of excess weight and obesity. Although some controversy exists, FM percentages that exceed 25% in males and 30% in females are the most commonly used cut-off points to define excess of adiposity in adults [40]. However, there is no consensus regarding the cut-off points in children. In this study, a statistical criteria was used, based on the standardized scores, as proposed by the WHO, with the anthropometric and weight-height indicators [5], and the IOTF criteria [26]. In this study, the classification with both criteria coincides for the normal FM groups (Table 1 and Fig 1). The IOTF criteria classified many more children as obese, given that its cut-off point for obesity is much lower (Z = 1.64) than in the statistical criteria (Z = 2).

On the other hand, the fat mass index (FMI) is useful for the diagnosis of excess of weight in adolescents, having an excellent relationship between sensitivity and specificity for this indicator, as demonstrated by Alvero-Cruz et al. [41], but there are no reference values for the population under the age of 12. The relationship between the different levels of %FM, as explained previously, was also observed in subjects under the age of 12 and in children over the age of 12 in this study (Table 2 and Fig 2).

Few studies have examined the concordance between BMI and %FM in children. Wohlfahrt-Veje C et al. [42] measured body fat in 2,647 healthy children using DXA and revealed that in children with normal BMI, the %FM was quite variable (between 6% and 37%), and the sensitivity and specificity of the BMI depended in large part on the cut-off points used to classify the excess of adiposity. That is, using the criteria established for the %FM (by DXA) the BMI identified 148 out of 195 children with excess of FM, whereas when using the adult cut-off points for %FM, the sensitivity of the BMI decreased, and only 97 out of 195 were classified as obese via DXA. In this study, the analysis of concordance confirmed that BMI does not reflect body composition (Fig 3 and Tables 3 and 4), so that 20 subjects of the total sample (12%) with a normal FM, were classified as having excess body mass by the BMI.

Given the limitations mentioned, a vectorial analysis of bioimpedance was conducted. Although few studies have examined the applications of this type of BIA in children, reference patterns have been obtained from healthy youth [19,43]. This technique appears to be promising in Pediatrics, given that the raw, standardized electric data is directly used, based on height (R/H and Xc/H), regardless of body weight and without the need to apply predictive models. Furthermore, it has been confirmed that this method is useful in clinical settings to assess variations in hydration and nutritional state in obese subjects [44]. Validation studies carried out in adults have revealed that the vectors of subjects with abnormal values of tissue impedance are situated outside of the tolerance ellipse of 75% [45].

The comparison between the groups in the confidence ellipses of 95% reveal that the classification based on the Z-BMI and the Z-%FM applying the statistical criteria significantly discriminated between the subjects classified as normal, those who were overweight and those who were obese (Fig 4a and 4b). However, the overlapping of the ellipses in the classification of the Z-%FM with the IOTF criteria reveal that these cut-off points overlay the categories of overweight and obesity (Fig 4c). And the same is observed in children under the age of 12 (Fig 6). In children over the age of 12, only the classification of BMI and the fat mass index discriminate between the ranks of obesity and overweight (Fig 5a) and obesity compared to non-obesity (Fig 5d), respectively. Therefore, the application of a statistical criteria better classifies the adiposity of the total sample and the group of children under the age of 12, while in children over the age of 12, the fat mass index is a better indicator.

To study the position of the individual vectors of participants in the study, the RXc-score-graph was used. The comparison between the nutritional classification both based on Z-BMI as well as Z-%FM and in those over the age of 12 also that of the FMI, reveal some major differences (Fig 7). As expected, an in accordance with previous studies carried out in children and adults with overweight or obese [45,46], most of the individual vectors are situated in the left lower quadrant, where subjects having an excess of body mass in comparison to their free-fat mass are placed, suggesting an excess of adiposity. However, 19.8%, 23.9% and 15.2% of the individual vectors are situated outside of the tolerance ellipse of 95% in the total sample, in the group of children under the age of 12 and in the group of children over the age of 12, respectively, indicating abnormal impedance vectors (Fig 7). These findings are similar to those documented in most of the studies carried out in adults, in which it has been seen that a high percentage of the individual vectors of obese subjects are situated outside of the confidence ellipse of 75% [44,47].

Despite the advantages of BIVA, this method does not permit assessment of body composition according to the bi-compartmental model, as documented by Buffa et al. [48]. Although there is no general agreement on this regard, some authors have confirmed that the two modalities of BIA similarly classified subjects [46], and other results were inconsistent [49]. Studies on adults using DXA as a reference technique to assess body composition have revealed that BIVA is capable of discriminating between subjects with different %FM, although with a reduced precision [50].

In adult subjects, it has been observed that the displacement to the right of the individual vectors located on the lower quadrant is more suggestive of a fluid overload than an accumulation of fat mass [44]. The displacement of the individual vectors toward the inferior-right quadrant (cachexia), parallel to the lower axis of the tolerance ellipses, has been associated with a decrease in the values of reactance, suggesting a loss of cell mass and, perhaps, alterations in the integrity of the cell membrane. This pattern may also be related to an increase in the total body water/extracellular water ratio. Although there are no studies that reproduce these findings in childhood, our results indicate similar behavior in children.

Other types of BIVA, such as the specific-BIVA recently developed in adults and elderly [50,51], might improve the study of body composition in childhood. This method is different from the classic BIVA (until now called BIVA) in that the electric variables (R and Xc) standardized based on height, are corrected, according to Ohm’s law, by a factor that represents the transversal section of the body segments (arm, waist, leg). In studies conducted in adults and elderly individuals, it was observed that compared to DXA, the specific-BIVA can distinguish between individuals with different levels of FM.

This study presents certain limitations. Although the sample size is not small, a larger sample will allow obtain more accurate results. On the other hand, the data collection was done retrospectively, between 2003 and 2015. However, the aim of the study was to assess the efficacy and usefulness of bioelectrical impedance vector analysis on the study of body composition in a group of children with overweight and obesity, so the age of the data is irrelevant. Finally, a gold standard technique has not been used to assess the fat mass, but the technique used (bioimpedance analysis) is one of the most used in clinical practice; so, we think that the results presented are useful for the monitoring of the nutritional status in clinical settings.

In conclusion, the vectorial analysis of bioimpedance reflects differences in the bioelectrical patterns of children classified with excess weight and obesity based on BMI and distinct levels of FM ranked with a statistical criteria. Moreover, in children over the age of 12, the BIVA clearly discriminates between obese and non-obese individuals, according to the fat mass index. Despite the limitations of the BIVA in obese children, the use of the RXc-score-graph allows the monitoring of the nutritional status over the years and the changes associated with body composition in a fast and simple manner. Furthermore, this modality is not affected by errors derived from the use of the traditional BIA prediction equations when the individual vectors are outside of the tolerance ellipse of 75%. So, the advantages of the BIVA include its ability to determine in which children the quantitative estimation of the body compartments based on traditional BIA will be reasonably precise.

## Supporting information

S1 Dataset Obese children xlsx. (XLSX)
